# Perioperative Use of IgM-Enriched Immunoglobulins in Liver Transplantation Recipients at High Risk for Infections: A Preliminary Study

**DOI:** 10.3390/jcm13164965

**Published:** 2024-08-22

**Authors:** Erika Roat, Martina Tosi, Irene Coloretti, Filippo Bondi, Giovanni Chierego, Stefano De Julis, Marta Talamonti, Emanuela Biagioni, Stefano Busani, Stefano Di Sandro, Erika Franceschini, Gian Piero Guerrini, Marianna Meschiari, Fabrizio Di Benedetto, Cristina Mussini, Massimo Girardis

**Affiliations:** 1 Anesthesia and Intensive Care Unit, University Hospital of Modena, University of Modena and Reggio Emilia, L.go del Pozzo 71, 41125 Modena, Italy; erikaroat@gmail.com (E.R.); tosimartina@gmail.com (M.T.); irenecoloretti@gmail.com (I.C.); bondipippo@gmail.com (F.B.); g.chierego@gmail.com (G.C.); dejulis@hotmail.com (S.D.J.); marty.talamonti@gmail.com (M.T.); emanuela.biagioni@gmail.com (E.B.); stefano.busani@unimore.it (S.B.); 2 Hepato-Pancreato-Biliary Surgery and Liver Transplantation Unit, University Hospital of Modena, University of Modena and Reggio Emilia, 41121 Modena, Italy; stefano.disandro@unimore.it (S.D.S.); gianpiero.guerrini@unimore.it (G.P.G.); fabrizio.dibenedetto@unimore.it (F.D.B.); 3 Infectious Disease Unit, University Hospital of Modena, University of Modena and Reggio Emilia, 41121 Modena, Italy; ericafranceschini0901@gmail.com (E.F.); meschiari.marianna@aou.mo.it (M.M.); cristina.mussini@unimore.it (C.M.)

**Keywords:** liver transplantation, postoperative infections, hypogammaglobulinemia, intravenous immunoglobulin therapy, IgM

## Abstract

**Background**: Infections frequently occur after orthotopic liver transplantation (OLT) and are associated with increased mortality. In 2018, we introduced perioperative administration of intravenous immunoglobulin enriched in IgM as an optional therapy in recipients at a high risk of infection. This preliminary study evaluated whether this preparation reduced infections in the early post-transplantation period. **Methods**: Adult patients with a high risk of postoperative infections who underwent OLT between January 2014 and December 2021 in our center were included in the study. The primary outcome was the occurrence of new postoperative bacterial and fungal infections within the first 30 days after OLT. **Results**: Ninety recipients at a high risk of postoperative infections who underwent OLT were included, of whom 51 (57%) received IgM preparation. Patients treated and not treated with IgM were similar in terms of demographics, model of end-stage liver disease score, and risk factors for postoperative infections. The occurrence of new infections was lower (absolute risk reduction (ARR) 21.2%; *p* = 0.038) in patients who received IgM than in those who did not. Multivariate analysis adjusted for confounders (OR 0.348; *p* = 0.033) and propensity score-based matching analysis (ARR 21.2%, *p* = 0.067) confirmed an association between IgM preparation and lower occurrence of postoperative infections. The 90-day mortality rate was lower (ARR 13.4%, *p* = 0.018) in patients who received IgM preparation. **Conclusions**: In OLT recipients at high risk for infections, perioperative administration of an IgM-enriched preparation seems to reduce the development of new infections within the first 30 days after OLT.

## 1. Introduction

Postoperative infections are significant causes of morbidity and mortality in solid organ transplantation, such as orthotopic liver transplantation (OLT), where over 50% of recipients experience infections [1,2,3,4]. Several risk factors for postoperative infections have been identified, depending on recipient and donor conditions and intraoperative and postoperative events [5]. Recipient risk factors are the most frequent and include advanced age, previous transplantation, diabetes, chronic renal insufficiency, exposure to antibiotics within 30 days before transplantation, and previous admission to the intensive care unit (ICU) [6]. These two latter factors are also related to an increased risk of infections sustained by difficult-to-treat microorganisms, which further increases the risk of recipient mortality. Notably, the high risk of postoperative infections frequently leads to delayed transplantation and consequently, increased mortality of candidates on the waiting list. 

Specific strategies to decrease postoperative infections are commonly adopted in OLT recipients, including antibiotic and antifungal prophylaxis, appropriate donor selection, and modulation of immunosuppressive regimens. In addition to immunosuppressors, several other factors may impair the inflammatory immune response in recipients. As in the case of OLT, extensive surgical procedures may induce a robust inflammatory response followed by an anti-inflammatory compensative phase that may lead to postoperative immune paralysis [7,8]. Cirrhosis is commonly associated with alterations in both innate (altered toll-like receptor expression and function, reduced phagocytic capacity of Kupfer cells) and adaptive immune (B cells dysfunction, chronic activation, and subsequent exhaustion of T cells) responses that lead to deep immune dysfunction, namely cirrhosis-associated immune dysfunction syndrome, which makes cirrhotic patients at high risk of infection before and after OLT [9,10]. To date, no specific therapies have been proposed to support the immune system during the postoperative phase of transplantation. 

In the immune response, immunoglobulins (Ig) play multiple key roles in the clearance of pathogens and toxins and modulation of antigen-presenting cell and lymphocyte activities [11]. For their pleiotropic effects, intravenous polyclonal immunoglobulins (IVIg) are used in many immune-mediated diseases and to treat infections and sepsis [12,13]. We hypothesized that perioperative use of intravenous immunoglobulins in OLT recipients might provide clinical benefits by supporting the immune response. Therefore, in our internal clinical protocol, we introduced the option of administering intravenous Ig in the perioperative period of OLT in recipients at high risk for infections.

In this retrospective observational study, we evaluated whether the perioperative use of IVIg with an IgM-enriched preparation could reduce infections in the early post-transplantation period in OLT recipients at high risk for post-operative infections.

## 2. Materials and Methods

This observational retrospective study included adult patients at high risk of postoperative infections who underwent OLT between January 2014 and December 2021 at the University Hospital of Modena. The patients were included in the study if one or more of the following risk factors for postoperative infections [6,14] were present: ongoing infection requiring specific antibiotic therapy on the day of transplantation, previous infection within 30 days before OLT, previous colonization by multidrug-resistant (MDR) bacteria and admission to ICU within 30 days before OLT. All the research was conducted in accordance with the Declaration of Helsinki and Istanbul. The study was approved by the Institutional Ethics Committee of Area Vasta Emilia Nord (EC AVEN) (n = 215, 8 July 2014), and informed consent was obtained from all participants.

In 2018, perioperative administration of IVIg enriched in the IgM component (Pentaglobin-Biotest, Dreieich, Germany) was included as an optional adjunctive therapy in the internal protocol for perioperative management of liver transplant recipients who presented with the aforementioned risk factors for postoperative infections. The internal protocol was discussed and shared with the multidisciplinary liver transplantation team at our center. The decision to use an IgM-enriched preparation was left to the attending anesthesiologist, who based their choice on the protocol indications and personal evaluation of the patient’s risk. In cases of uncertainty, the team’s reference persons were consulted. The IgM preparation was administered in continuous intravenous infusion at a dosage of 6.25 mg/kg/h, starting with antibiotic administration before surgical incision and continuing for 24 h (total dose 150 mg/kg). In case of severe bleeding during surgery [15,16] the infusion of IgM preparation was stopped and restored at the end of bleeding by extending the infusion beyond 24 h up to the completion of the 150 mg/kg dose. The perioperative antibacterial and antifungal prophylaxis strategies did not change during the study period (see Supplemental Material). In patients colonized by multidrug-resistant pathogens (MDR), the prophylaxis strategy was adjusted based on the sensitivity pattern of the colonizing microorganisms (non-standard antibiotic prophylaxis). The standard immunosuppressive regimens remained similar throughout the study period in patients with and without ongoing infections. Likewise, the other standard surgical and anesthesiologic procedures remained relatively unchanged during the study period.

Demographics, clinical data, and Ig plasma levels collected 24 h before and 24 h after transplantation were obtained from the clinical charts and laboratory data. The primary outcome was the occurrence of new postoperative bacterial and fungal infections within the first 30 days after OLT and the secondary outcomes were the incidence of new respiratory, urinary, and rectal colonization by multidrug-resistant pathogens (MDR) in the first 30 days, intensive care and hospital-free days at days 30 and 90 after OLT, and the mortality rate at days 30 and 90 after OLT. Post-transplant infections were defined according to the criteria defined in international guidelines, new respiratory infections only included pneumonia defined as the presence of a new persistent infiltrate observed at the chest radiograph or computed tomography scan associated (at least one) with the worsening of oxygenation, purulent bronchial secretions, leukocytosis, and fever, and the presence of potentially pathogenic microorganisms in culture from tracheal aspirate and bronchoalveolar lavage. Bacteremia was defined as the presence of pathogenic microorganisms in the blood cultured from peripheral and/or central venous lines. Isolation of coagulase-negative Staphylococci in a single blood culture was not considered as bacteremia. Regarding postoperative intra-abdominal infections various classifications have been proposed in literature, we included postoperative intra-abdominal abscess i.e., a postoperative collection of infected fluid within the abdominal cavity and secondary peritonitis [17,18,19]. To minimize bias when determining postoperative infections, an infectious disease specialist (EF) and a well-experienced intensivist (BM) controlled and revised the clinical and microbiological data of the studied patients. Only microbiologically proven infections were considered in this study. MDR microorganisms were defined as isolates that were non-susceptible to at least one agent in the three antimicrobial categories listed in the standard definitions for acquired resistance [20]. 

Non-parametric and χ^2^ tests were used as appropriate to compare demographic and baseline values and outcomes in patients receiving and not receiving IgM preparation. All results are expressed as medians and interquartile ranges for continuous variables and as frequencies and percentages for categorical variables. We used a multivariate logistic model to evaluate the relationship between postoperative infections within 30 days and the perioperative use of IgM preparation, including variables with a *p*-value < 0.1 in the unadjusted analysis. 

To address the potential impact of confounding variables, we conducted a secondary analysis using propensity score matching for the patient cohorts. The estimation of the individual propensity to receive IgM treatment was accomplished through the utilization of a multivariable logistic regression model, which incorporated the MELD score and the pertinent risk factors for study inclusion (ongoing infection requiring specific antibiotic therapy the day of transplantation, previous infection within 30 days before OLT, previous colonization by MDR bacteria, admission to ICU within 30 days before OLT); the nearest-neighbor method with a caliper of 0.2 was applied to the propensity score matching analysis (1:1). 

In the subgroup of patients with available data, the plasma levels of Ig measured 24 h before and 24 h after transplantation were compared between patients with and without new infections during the first 30 days after transplantation as well as between individuals who received IgM therapy and those who did not. SPSS version 22.0 package (SPSS Inc., Chicago, IL, USA) was used to perform statistical analysis.

## 3. Results

From January 2014 to December 2021, a total of 456 patients underwent orthotopic liver transplantation (OLT). Based on the risk factors for postoperative infections, 90 patients (20%) were included in the study, of whom 51 (57%) received IgM preparation in the perioperative period. Patients treated and not treated with IgM were similar in demographics, model of end-stage liver disease (MELD) score, and causes of end-stage liver disease (ESLD) (Table 1). Most patients had more than one risk factor for postoperative infections that was similar between patients treated and not treated with IgM. The percentage of infections sustained by MDR microorganisms before OLT was significantly higher (*p* = 0.021) in patients who received (35.3%) than in those who did not receive IgM (10.3%). 

The occurrence of new bacterial and fungal infections within day 30 after OLT was lower (absolute risk reduction 21.2%, *p* = 0.038) in patients who received compared to those who did not receive IgM preparation (Table 2). Gram-negative bacteria accounted for 45% of the infections, while Gram-positive bacteria accounted for 37% and fungi for 18% (*Candida* spp. 11%, *Aspergillus* F. 7%), specifically *Candida* spp. (Table S1). The incidence of new colonization by MDR microorganisms, ICU- and hospital-free days, and the mortality rate at day 30 were similar between the two groups of patients. In patients who received IgM, the 90-day mortality was lower (absolute risk reduction 13.4%, *p* = 0.018) than that in patients who did not receive IgM preparation infusion (Table 2). Of the seven patients who died within the initial 90 days, all except one experienced recurrent infection episodes during the postoperative period. These infections were either primary or related to surgical complications and/or graft dysfunction.

Multivariate analysis adjusted for variables with a *p*-value < 0.1 in the unadjusted analysis (MELD score and ICU admission before OLT) showed that the use of IgM preparation reduced (OR 0.348, CI 0.132–0.919; *p* = 0.033) independently of the occurrence of infections in the first 30 days after transplantation (Table 3).

Sixty-six patients (IgM, n = 33; no IgM, n = 33) out of the 90 studied (70%) were included in the propensity score-based analysis, and all confounding factors were well-balanced between the two groups (see Supplemental Material, Table S2). The occurrence of new bacterial and fungal infections within 30 days after OLT was lower in matched patients who received the IgM preparation (27.3%) than in those who did not (48.5%), with an absolute risk reduction of 21.2% (*p* = 0.067) (see Supplemental Material, Table S3).

**Figure 1:**
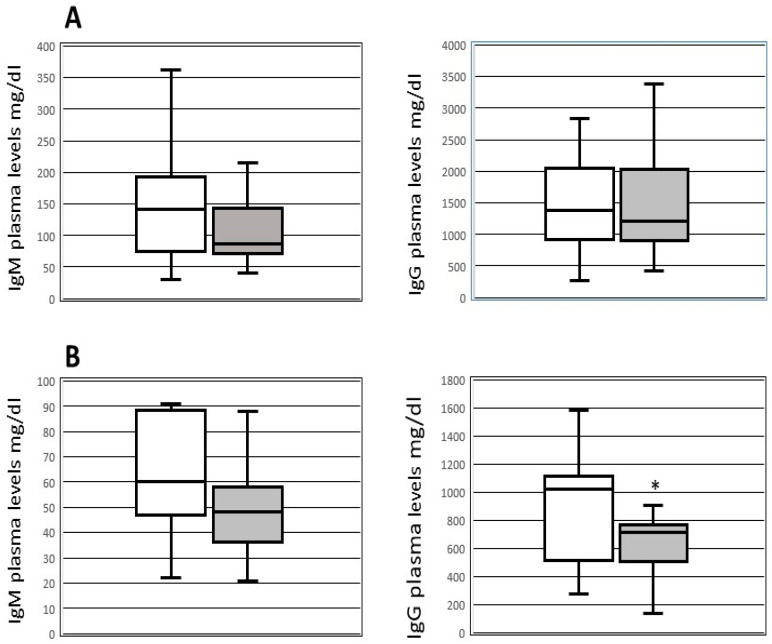
**Box-plot of plasmatic levels of IgG and IgM before and after transplantation.** IgM and IgG plasmatic levels pre-OLT (upper part—(**A**)) and within 48h after OLT (lower part—(**B**)) in patients with (black boxes) and without (white boxes) new infections after transplantation. * *p* < 0.039 compared to patients without infections.

In the subgroup of patients with available data on plasma Ig levels (36 patients, 26 receiving and 10 not receiving IgM therapy) before (within 24 h) and after transplantation (within 24 h), we did not detect differences in the levels of pre-OLT immunoglobulin between patients who developed (14 patients) and those who did not develop (29 patients) infections. After OLT, the IgG plasma levels were lower (*p* = 0.039) in patients with the occurrence of new infections (median 715; IQR 505–765 mg/dL) than in those without infections (median 1024; IQR 524–1106 mg/dL) (Figure 1). Furthermore, after transplantation patients who received IgM therapy exhibited higher levels of IgG (median 524 mg/dL, IQR 377–794 mg/dL) and IgM (median 39 mg/dL, IQR 29–50 mg/dL) (*p* < 0.05) compared to those who did not receive IgM therapy (median IgG 790 mg/dL, IQR 584–1094 mg/dL; median IgM 60 mg/dL, IQR 48–88 mg/dL). Patients receiving IgM preparation did not show adverse reactions related to immunoglobulins intravenous infusion. 

## 4. Discussion

Our preliminary study indicated that the perioperative use of intravenous immunoglobulin therapy with a preparation enriched in IgM seems to reduce by 20% the occurrence of new bacterial and fungal infections after OLT transplantation, with an improvement in the survival rate at day 90. 

Various factors contribute to the high risk of OLT recipients for postoperative infectious complications [4,6]. Moreover, pre-transplantation infections caused by multidrug-resistant (MDR) microorganisms are linked to an increased likelihood of post-transplantation infections and elevated mortality rates [21,22]. During the study period at our center, it was observed that approximately 20% of patients undergoing OLT had risk factors, and the characteristics of these patients were consistent between those treated and not treated with IgM preparations, with the exception of pre-OLT infections caused by MDR bacteria, which were more prevalent in patients who received IgM than in those who did not. It is notable that despite the higher prevalence of MDR infections before OLT, the rate of patients with postoperative infections was lower in those treated with IgM preparation than in those not treated. As anticipated, the reduction of new infections was associated with a benefit in the survival rate at day 90 in patients who received the IgM preparation.

Few studies have examined the use of IVIg therapy in solid organ transplantation. After solid organ transplantation, hypogammaglobulinemia, defined as IgG levels below 650 or 700 mg/dL, is common after heart, lung, and kidney transplantation [23,24] and is associated with an increased risk of infections. In heart recipients with HGG, the use of IVIg decreased the infection rate and mortality, whereas no effects were observed in lung and intestinal recipients with HGG [25,26,27]. In OLT recipients, HGG is less frequent than in other solid organ transplantations [28]. Doron et al. [25] reported an HGG incidence of 26% in the first five days after transplantation, with a strong association with high mortality risk. In contrast, other studies have observed a relationship between HGG and infection risk after OLT in children and living donor transplantation [29]. Although available in only half of the studied population, our data confirmed the association between early postoperative low levels of immunoglobulin subtype G and increased infection risk. Our research did not examine the advantages of IVIg therapy beyond the immediate perioperative period. Nonetheless, HGG can still develop after the postoperative period has ended [23], and in such cases, IVIg therapy might be taken into account in order to lessen the likelihood of infections or as a supplementary therapy for patients with severe infections.

Immunoglobulins hold considerable importance in preventing infections by neutralizing or eradicating pathogens and toxins, as well as by stimulating the activity of immune cells to enhance the immune response. Additionally, they play a vital role in preserving overall immune health by regulating and controlling immune responses [11]. Several research studies have underscored the protective role of immune and natural immunoglobulins subtype M (IgM) against viral, bacterial, fungal, and parasitic infections [30]. This subtype of immunoglobulins has a distinct structure that enables it to mediate the agglutination of invading pathogens. Moreover, its polyreactivity allows for binding to different structures on the same pathogen, enhancing the neutralization of the invading organism. Moreover, numerous studies in mice have highlighted the importance of natural IgM in resistance to fungal infections through a variety of mechanisms, including the recruitment of macrophages and phagocytosis of fungi, the assistance in the recognition of fungal antigens by dendritic cells and their migration to lymph nodes, and the primary role of antibodies in generating memory anti-fungal immunity [31,32].

Surgery alone may contribute to immune dysfunction, especially in cases of long operative times and severe intraoperative bleeding. Notably, in OLT recipients, endotoxin levels appear to be elevated throughout the transplantation procedures, with the highest systemic levels measured after vascular anastomosis in patients who developed postoperative complications [33]. These factors lead us to initiate IgM infusion before surgery to support antibiotics and mitigate the effects of surgery, endotoxins, and bacterial translocation on the immune response. 

The limitations of our study include its design, small sample size, brief follow-up, and failure to analyze perioperative factors that may affect infection risk such as blood transfusions, time of the procedure, incidence of graft dysfunction, renal dysfunction, and time of mechanical ventilation. Additionally, the optional use of IgM was left to the discretion of the attending anesthesiologists, which may have introduced unmeasurable bias. However, we attempted to overcome these limitations by utilizing multivariate analysis and a propensity score-based patient-matching approach. Moreover, the total dose of IgM preparation (150 mg/kg for 1 Day) was based on the concept of prophylactic use; therefore, we reduced the standard dosage and the duration of IgM therapy (250 mg/kg/day for 3 days). A proper phase II trial based on different IgM preparation dosages, or a strategy based on the patient’s Ig plasma levels before transplantation, as in the ongoing trial on septic shock [34], should be considered for defining the appropriate dose of IgM preparation. 

In summary, our study findings indicate a correlation between postoperative HGG and increased risk of infection in OLT recipients. Perioperative administration of IVIg with an IgM-enriched preparation seems to mitigate new infections and improve survival in high-risk OLT recipients. It is essential to emphasize that the present findings should be approached prudently and may not be universally applicable beyond the scope of the present investigation. Additional well-constructed clinical trials are warranted to assess the implications of perioperative administration of IgM preparation more comprehensively on infections, survival, and the most favorable timing and dosage for this therapeutic approach. 

## Figures and Tables

**Table 1:** Demographics, severity score, and pre-transplantation infective condition in patients treated and not treated with IgM enriched preparation.

	Total (n = 90)	No IgM (n = 39)	IgM (n = 51)	*p*-Value
Age (years; median-IQR)	56 (50–63)	57 (53–63)	55 (50–63)	0.194
Female (n, %)	27 (30)	11 (28.2)	16 (31.4)	0.744
Diabetes (n, %)	23 (25.6%)	10 (25.6%)	13 (25.5%)	0.987
MELD score (median-IQR)	22 (12–29)	22 (12–29)	20 (12–29)	0.625
Causes of ESLD (n, %)				
*Hepatocellular carcinoma*	20 (22.2)	10 (25.6)	10 (19.6)	0.495
*HBV-related*	10 (11.1)	5 (12.8)	5 (9.8)	0.652
*HCV-related*	21 (23.3)	11 (28.2)	10 (19.6)	0.339
*Alcoholoic*	38 (42.2)	16 (41.0)	22 (43.1)	0.841
*Other causes*	41 (45.6)	14 (35.9)	27 (52.9)	0.128
Non -standard antibiotic prophylaxis (n, %)	83 (92.2)	34 (87.2)	46 (90.2)	0.118
Anti-fungal prophylaxis (n, %)	39 (43.3)	19 (48.7)	20 (39.2)	0.258
Risk Factors for post-operative infections (n, %)				
*Ongoing Infections*	37 (41.1)	13 (33.3)	24 (47.1)	0.191
*Infections 30 days before OLT*	53 (58.9)	23 (59)	30 (58.8)	0.982
*Colonization MDR 30 days before OLT*	59 (65.6)	27 (69.2)	32 (62.7)	0.374
*ICU admission 30 days before OLT*	18 (20)	10 (25.6)	8 (15.7)	0.246

Abbreviations: n, number; IQR, interquartile range; OLT, orthotopic liver transplantation; MELD, model of end-stage liver disease; ESLD: end-stage liver disease; HBV, hepatitis B virus; HCV, hepatitis C virus; IgM, IgM-enriched polyclonal immunoglobulins preparation; ICU, intensive care unit; MDR: multidrug resistance micro-organisms. Other causes included: autoimmune diseases, re-transplant for complications, hemochromatosis, and Budd-Chiari syndrome.

**Table 2:** New infections in the first 30 days after transplantation, intensive care unit and hospital-free days, and mortality at day 30 or 90 after transplantation in patients treated and not treated with IgM enriched preparation.

	Total (n = 90)	No IgM (n = 39)	IgM (n = 51)	*p*-Value
New Infections 30 days after-OLT (n, %)	33 (36.7)	19 (48.7)	14 (27.5)	0.038
New respiratory tract infections 30 days after-OLT (n, %)	16 (17.8)	8 (20.5)	8 (15.7)	0.123
New abdominal infections 30 days after-OLT (n, %)	14 (15.6)	9 (23)	5 (9.8)	0.140
New primary blood stream infections 30 days after-OLT (n, %)	5 (5.6)	4 (10.3)	1 (2)	0.089
New other infections 30 days after-OLT (n, %)	4 (4.4)	2 (5.1)	2 (3.9)	0.783
MDR infections 30 days after OLT (n, %)	10 (11.1)	6 (15.4)	4 (7.8)	0.208
New colonization by MDR 30 days after OLT (n, %)	24 (26.7)	12 (30.8)	12 (23.5)	0.442
New septic shock 30 days after-OLT (n, %)	5 (5.6)	3 (7.7)	2 (3.9)	0.421
ICU free days at day 30 after OLT (median, IQR)	27 (24–28)	26 (25–27)	27 (24–28)	0.231
New AKI requiring CRRT 30 days after-OLT (n, %)	5 (5.6)	2 (5.1)	3 (5.9)	0.900
Re-laparotomy 30 days after-OLT (n, %)	11 (12.2)	4 (10.3)	7 (13.7)	0.650
Hospital free days at day 90 (median, IQR)	69 (56–77)	71 (63–76)	67 (55–77)	0.503
Mortality at day 30 post OLT (n, %)	1 (1.1)	1 (2.6)	0 (0)	0.252
Mortality at day 90 post OLT (n, %)	7 (7.8)	6 (15.4)	1 (2)	0.018

Abbreviations: n, number; OLT, orthotopic liver transplantation; MDR, multi-drug resistant; IgM, IgM-enriched polyclonal immunoglobulins preparation; ICU, intensive care unit.

**Table 3:** Demographics, severity score, and pre-transplantation conditions of patients with or without infections during the first month after transplantation. The results of unadjusted and adjusted analyses are also reported.

	No Infections Post-OLT (n = 57)	Infections Post-OLT (n = 33)	Unadjusted OR (95% CI); *p*-Value	Adjusted OR (95% CI); *p*-Value
Age; (years; median, IQR)	57 (51–63)	56 (50–62)	1.002 (0.958–1.048); 0.944	
Sex, female (n, %)	19 (33.3)	8 (24.2)	0.640 (0.243–1.685); 0.384	
MELD score (median, IQR)	18 (11–29)	25 (12–31)	1.054 (1.004–1.106); 0.034	1.034 (0.979–1.091); 0.229
Causes of ESLD (n, %)				
*Hepatocellular carcinoma*	11 (19.3)	9 (27.3)	1.568 (0.571–4.305); 0.381	
*HBV-related*	6 (10.5)	4 (12.1)	1.172 (0.306–4.499); 0.817	
*HCV-related*	15 (26.3)	6 (18.2)	0.622 (0.215–1.801); 0.379	
*Alcoholic*	23 (40.4)	15 (45.5)	1.232 (0.518–2.928); 0.637	
*Other causes*	26 (45.6)	15 (45.5)	0.994 (0.420–2.350); 0.988	
Risk Factors for infections (n, %)				
*Ongoing Infections*	22 (38.6)	15 (45.5)	1.326 (0.556–3.159); 0.524	
*Infections 30 days before OLT*	32 (56.1)	21 (63.6)	1.367 (0.566–3.301); 0.486	
*Colonization MDR 30 days before OLT*	39 (68.4)	20 (60.6)	0.808 (0.327–1.992); 0.643	
*ICU admission 30 days before OLT*	6 (10.5)	12 (36.4)	4.857 (1.610–14.649); 0.003	3.496 (1.002–12.205); 0.050
IgM (n, %)	37 (64.9)	14 (42.4)	0.378 (0.205–0.700); 0.002	0.348 (0.132–0.919); 0.033

Abbreviations: n, number; IQR, interquartile range; OLT, orthotopic liver transplantation; MELD, model of end-stage liver disease; ESLD: end-stage liver disease; HBV, hepatitis B virus; HCV, hepatitis C virus; IgM, IgM-enriched polyclonal immunoglobulins preparation; ICU, intensive care unit. Other causes included: autoimmune diseases, re-transplant for complications, hemochromatosis, and Budd-Chiari syndrome.

## Data Availability

Data is available on request due to privacy/ethical restrictions.

## Supplementary Materials

The following supporting information can be downloaded at: https://www.mdpi.com/article/10.3390/jcm13164965/s1, Table S1: Microorganisms causing infections in the first 30 days after OLT in patients treated and not treated with IgM enriched preparation; Table S2: Demographics, severity score and pre-transplantation infective condition, in a subpopulation of 66 patients obtained by propensity score based matching, treated and not treated with IgM enriched preparation; Table S3: New bacterial infections in the first 30days after transplantation, intensive care unit and hospital free days, and mortality at day 30 and 90 after transplantation, in a subpopulation of 66 patients obtained by propensity score based matching, treated and not treated with IgM enriched preparation, treated and not treated with IgM enriched preparation.

## Abbreviations

OLT	orthotopic liver transplantation
IVIg	polyclonal standard IgG
ICU	intensive care unit
IgM	IgM-enriched polyclonal immunoglobulins product
MELD	model of end stage liver disease
ELSD	end stage liver disease
ICU	intensive care unit
Ig	immunoglobulins
HGG	hypogammaglobulinemia
MDR	multi drug resistant
HCC	hepatocellular carcinoma
HBV	hepatitis B virus
HCV	hepatitis C virus
LD	liver disease
