# Supplementary material for: Perioperative Use of IgM-Enriched Immunoglobulins in Liver Transplantation Recipients at High Risk for Infections: A Preliminary Study
Source: J Clin Med. 2024 Aug 22;13(16):4965. doi: 10.3390/jcm13164965 (PMC11355682; doi:10.3390/jcm13164965)
Supplement: Supplementary file 1 [file jcm-13-04965-s001.zip › jcm-3066209-supplementary.pdf]

SUPPLEMENTAL MATERIAL

Antibiotic and antifungal prophylaxis in Liver transplant recipients

Standard antibiotic prophylaxis for LT is performed with ev cefotaxime (Cefotaxime, Mylan Generics, Italy) 2 gr plus ev ampicillin (Ampicillina, Biopharma, Italy) 2 gr at anesthetic induction, with a supplementary dose administration after 3 and 6 hours during the surgical procedure and then every 8 hours for 3 days) . For patients allergic to penicillin standard prophylaxis is ev ciprofloxacin (ciprofloxacin, Mylan Generics, Italy) 400 mg at anesthetic induction, then every 6 hours during the procedure and then every 12 hours, plus ev clindamycin (Dalacin, Pfizer, Italy) 600 mg at anesthetic induction, then every 6 hours during the procedure and then every 8 hours, both for 3 days.

Patients with a pre-LT surveillance rectal swab positive for a pathogen resistant to standard prophylaxis (either MDR gram negative or VRE) received an individualized peri-operative anti-bacterial prophylaxis for 3 days, based on the bacteria susceptibility test.

Other prophylaxis

All patients received a PCP prophylaxis consisting in trimethoprim 160 mg and sulfamethoxazole 800 mg (Bactrim, Eumedica Pharmaceuticals, Germany) three times a week, as soon as oral administration could be tolerated and for the first 6-12 months after transplantation.

All patients with at least two risk factors for candidiasis received anti-fungal prophylaxis with fluconazole (Flucanazolo Kabi, Fresenius Kabi, Italy) (6mg/kg for a week). All patients with at least one risk factor for *Aspergillus* received liposomal amphotericin B (Ambisome, Gilead, Italy) 3mg/kg/die or anidulafungin 100 mg/die for a week.

LT recipients who were seronegative for CMV and received an organ from a CMV seropositive donor (D+/R-) had anti-viral prophylaxis with ganciclovir (Cymevene, Recordati, Italy) 5mg/kg/die or valganciclovir (Valganciclovir, Teva, Italy) 900mg/die for 3 months as soon as oral administration was possible. All the other patients received acyclovir as antiviral prophylaxis.

Table S1: Microorganisms causing infections in the first 30 days after OLT in patients treated and not treated with IgM enriched preparation.

	**TOTAL**	**NO IgM**	**IgM**	**p**
Microorganisms causing infections 30 days after OLT (n, %)	38	23	15	
Gram-positive microorganisms (n, %)	**17 (0,45)**	**10 (0,43)**	**7 (0,46)**	0, 847
Enterococcus spp. (n)	*7*	*4*	*3*	
Coagulase-negative staphylococci (n)	*5*	*3*	*2*	
Others (n)	*5*	*3*	*2*	
Gram-negative microorganisms (n, %)	**14 (0,37)**	**8 (0,35)**	**6 (0,40)**	0,749
Pseudomonas aeruginosa (n)	*5*	*3*	*2*	
Klebsiella spp (n)	*2*	*2*	*0*	
Escherichia coli (n)	*3*	*1*	*2*	
Others (n)	*4*	*2*	*2*	
Fungal infections (n, %)	**7 (0,18)**	**5 (0,22)**	**2 (0,14)**	0,514
Candida spp	**4**	**3**	**1**	
Aspergillus Fumigatus	**3**	**2**	**1**	

Abbreviations: IgM, IgM-enriched polyclonal immunoglobulins preparation

Table S2: Demographics, severity score and pre-transplantation infective condition, in a subpopulation of 66 patients obtained by propensity score based matching, treated and not treated with IgM enriched preparation.

	**TOTAL (n=66)**	**NO IgM (n=33)**	**IgM (n=33)**	**p-value**
Age (years; median- IQR)	56 (52-62)	57 (53-61)	55 (51-63)	0,437
Female (n, %)	17 (25,8)	9 (27,3)	8(24,2)	0,778
MELD score (median-IQR)	21 (12-29)	22 (12-29)	16 (11-27)	0,284
Causes of ESLD (n, %)				
Hepatocellular carcinoma	15 (22,7)	9 (27,3)	6 (18,2)	0,378
HBV-related	7 (10,6)	6 (18,2)	1 (3)	0,046
HCV-related	18 (27,3)	11 (33,3)	7 (21,2)	0,269
Alcholic	27 (40,9)	13 (39,4)	14 (42,4)	0,802
Other causes	26 (39,4)	9 (27,3)	17 (51,5)	0,044
Non -standard antibiotic prophylaxis (n, %)	83 (92,2)	29 (87,9)	31 (93,9)	0,392
Anti-fungal prophylaxis (n, %)	39 (43,3)	14 (42,4)	11 (33,3)	0,432
Risk Factors for post-operative infections (n, %)				
Ongoing Infections	37 (41,1)	13 (3339,4)	14 (42,4)	0,802

Infections 30 days before OLT	53 (58,9)	21 (63,6)	17 (51,5)	0,319
Colonization MDR 30 days before OLT	59 (65,6)	23 (69,7)	21 (63,6)	0,602
ICU admission 30 days before OLT	18 (20)	6 (18,2)	8 (24,2)	0,547

Abbreviations: n, number; IQR, interquartile range; OLT, orthotopic liver transplantation; MELD, model of end stage liver disease; ESLD: end stage liver disease; HBV, hepatitis B virus; HCV, hepatitis C virus; IgM, IgM-enriched polyclonal immunoglobulins preparation; ICU, intensive care unit; MDR: multidrug resistance micro-organisms. Other causes included: autoimmune diseases, re-transplant for complications, hemochromatosis, Budd- Chiari syndrome.

Table S3: New bacterial infections in the first 30days after transplantation, intensive care unit and hospital free days, and mortality at day 30 and 90 after transplantation, in a subpopulation of 66 patients obtained by propensity score based matching, treated and not treated with IgM enriched preparation, treated and not treated with IgM enriched preparation.

	**TOTAL (n=66)**	**NO IgM (n=33)**	**IgM (n=33)**	**p-value**
New Infections 30 days after-OLT (n, %)	25 (37,9)	16 (48,5)	9 (27,3)	0,067
MDR infections 30 days after OLT (n, %)	10 (15,2)	5 (15,2)	5 (15,2)	1
New colonization by MDR 30 days after OLT (n, %)	16 (24,2)	10 (30,3)	6 (18,2)	0,251
New Pneumonia 30 days after-OLT (n, %)	12 (18,8)	6 (18,2)	6 (18,2)	0,176
New abdominal infections 30 days after-OLT (n, %)	11 (16,7)	8 (24,2)	3 (9,1)	0,198
New primary blood stream infections 30 days after-OLT (n, %)	0 (0)	0 (0)	0 (0)	
New other infections 30 days after-OLT (n, %)	3 (4,5)	2 (6)	1 (3)	0,269
New fungal infections 30 days after-OLT (n, %)	5 (22,7)	5 (15,2)	0 (0)	0,020
New septic shock 30 days after-OLT (n, %)	1 (1,5)	1 (3)	0 (0)	0,321
ICU free days at day 30 after OLT (median, IQR)	27 (24-28)	26 (25-27)	27 (24-28)	0,424
New AKI requiring CRRT 30 days after-OLT (n, %)	3 (4,5)	1 (3)	2 (6)	0,534
Re-laparotomy 30 days after-OLT (n, %)	7 (10,6)	4 (12,1)	3 (9,1)	0,722
Hospital free days at day 90 (median, IQR)	66 (24-77)	68 (44-76)	66 (24-78)	0,928
Mortality at day 30 post OLT (n, %)	3 (4,5)	2 (6,1)	1 (3)	0,555

Mortality at day 90 post OLT (n, %)	7 (10,6)	5 (15,2)	2 (6,1)	0,230

Abbreviations: n, number; OLT, orthotopic liver transplantation; MDR, multi drug resistant; IgM, IgM-enriched polyclonal immunoglobulins preparation; ICU, intensive care unit.
